# Supplementary material for: Comprehensive Analysis of Bufadienolide and Protein Profiles of Gland Secretions from Medicinal Bufo Species
Source: Toxins (Basel). 2024 Mar 20;16(3):159. doi: 10.3390/toxins16030159 (PMC10975026; doi:10.3390/toxins16030159)
Supplement: Supplementary file 1 [file toxins-16-00159-s001.zip › toxins-2881695-supplementary.pdf]

Supplementary material: Comprehensive Analysis of Bufadienolide and Protein Profiles of Gland Secretions from Medicinal *Bufo* Species

Table S1. The top 10 proteins in terms of abundance for four *Bufo* species.

Species	Proteins	Function[1]
BgC	collagen alpha-1(VI) chain (*Xenopus laevis*)	Collagen VI acts as a cell-binding protein
	NEDD8-activating enzyme E1 regulatory subunit (*Xenopus laevis*)	NEDD8 activating enzyme activity
	transmembrane and immunoglobulin domain-containing protein 1 (*Bos taurus*)	May control cell-cell adhesion, cell migration and proliferation, cell morphology, and protects renal epithelial cells from oxidative cell injury to promote cell survival
	protein POF1B (*Mus musculus*)	actin filament binding
	alcohol dehydrogenase 1 (*Pelophylax perezi*)	alcohol dehydrogenase (NAD+) activity, zinc ion binding
	40S ribosomal protein S23 (*Ictalurus punctatus*)	structural constituent of ribosome
	phosphatidylinositol 5-phosphate 4-kinase type-2 gamma (*Xenopus tropicalis*)	1-phosphatidylinositol-4-phosphate 5-kinase activity, 1-phosphatidylinositol-5-phosphate 4-kinase activity, ATP binding
	thiopurine S-methyltransferase (*Rattus norvegicus*)	S-adenosyl-L-methionine binding, thiopurine S-methyltransferase activity
	vacuolar protein sorting-associated protein VTA1 homolog (*Bos taurus*)	Involved in the endosomal multivesicular bodies (MVB) pathway. MVBs contain intraluminal vesicles (ILVs) that are generated by invagination and scission from the limiting membrane of the endosome and mostly are delivered to lysosomes enabling degradation of membrane proteins, such as stimulated growth factor receptors, lysosomal enzymes and lipids. Thought to be a cofactor of VPS4A/B, which catalyzes disassembles membrane-associated ESCRT-III assemblies. Involved in the sorting and down-regulation of EGFR (By similarity)
	T-complex protein 1 subunit epsilon (*Macaca fascicularis*)	ATP binding, ATP hydrolysis activity, ATP-dependent protein folding chaperone
		beta-tubulin binding, G-protein beta-subunit binding, mRNA 3'-UTR binding, mRNA 5'-UTR binding, unfolded protein binding

BmS	arylsulfatase D (*Homo sapiens*)	arylsulfatase activity, metal ion binding
	src substrate cortactin (*Rattus norvegicus*)	actin filament binding, Arp2/3 complex binding, profilin binding, proline-rich region binding
	transcobalamin-2 (*Homo sapiens*)	cargo receptor ligand activity, cobalamin binding, metal ion binding
	phosphoglycerate mutase 2 (*Homo sapiens*)	2,3-bisphosphoglycerate-dependent phosphoglycerate mutase activity, bisphosphoglycerate mutase activity, hydrolase activity, identical protein binding, phosphoglycerate mutase activity
	galectin-4 (*Bos taurus*)	carbohydrate binding
	elongation factor 1-alpha 2 (*Rattus norvegicus*)	GTP binding, GTPase activity, protein kinase binding, translation elongation factor activity
	parvalbumin beta (*Xenopus laevis)*	calcium ion binding
	mesencephalic astrocyte-derived neurotrophic factor (*Bos taurus*)	growth factor activity, sulfatide binding
	ATP-binding cassette sub-family F member 1 (*Homo sapiens*)	ATP binding, ATP hydrolysis activity, ribosome binding, RNA binding, translation activator activity, translation factor activity, RNA binding
	translocon-associated protein subunit alpha (*Pongo abelii*)	TRAP proteins are part of a complex whose function is to bind calcium to the ER membrane and thereby regulate the retention of ER resident proteins. May be involved in the recycling of the translocation apparatus after completion of the translocation process or may function as a membrane-bound chaperone facilitating folding of translocated proteins (By similarity)
BaS	calcium-binding mitochondrial carrier protein SCaMC-1-B (*Xenopus laevis*)	ATP transmembrane transporter activity, ATP: inorganic phosphate antiporter activity, calcium ion binding
	tumor susceptibility gene 101 protein (*Homo sapiens*)	calcium-dependent protein binding, DNA binding, nuclear receptor coactivator activity, protein homodimerization activity, protein-containing complex binding, transcription corepressor activity, ubiquitin binding, ubiquitin protein ligase binding, virion binding
	catenin beta-1 (*Homo sapiens*)	alpha-catenin binding, cadherin binding, chromatin binding, disordered domain specific binding, DNA-binding transcription factor binding, enzyme binding, histone methyltransferase binding, I-SMAD binding, kinase binding, nuclear estrogen receptor binding, nuclear receptor binding, protein kinase binding, protein phosphatase binding, RNA polymerase II-specific DNA-binding transcription factor binding, SMAD binding, transcription coactivator activity, transcription coregulator binding, transcription corepressor

		binding, transmembrane transporter binding
	charged multivesicular body protein 2a (*Danio rerio*)	Probable core component of the endosomal sorting required for transport complex III (ESCRT-III) which is involved in multivesicular bodies (MVBs) formation and sorting of endosomal cargo proteins into MVBs. MVBs contain intraluminal vesicles (ILVs) that are generated by invagination and scission from the limiting membrane of the endosome and mostly are delivered to lysosomes enabling degradation of membrane proteins, such as stimulated growth factor receptors, lysosomal enzymes and lipids (By similarity)
	interferon-induced, double-stranded RNA-activated protein kinase (*Rattus norvegicus*)	ATP binding, double-stranded RNA binding, eukaryotic translation initiation factor 2alpha kinase activity,identical protein binding, non-membrane spanning protein tyrosine kinase activity, protein kinase activity, protein serine kinase activity
	hemoglobin subunit beta-2 (*Xenopus tropicalis*)	heme binding, metal ion binding, oxygen binding, oxygen carrier activity
	Alpha-actinin-1 (*Gallus gallus*)	actin filament binding, alpha-actinin binding, calcium ion binding, LIM domain binding, phosphoprotein binding, protein homodimerization activity, vinculin binding
	protein of unknown function	-
	COP9 signalosome complex subunit 3 (*Xenopus tropicalis*)	metal ion binding, metallopeptidase activity
	ubiquitin carboxyl-terminal hydrolase MINDY-3 (*Xenopus tropicalis*)	cysteine-type deubiquitinase activity, K48-linked deubiquitinase activity
BrS	nicotinamide N-methyltransferase (*Mus musculus*)	nicotinamide N-methyltransferase activity
	IgGFc-binding protein (*Homo sapiens*)	May be involved in the maintenance of the mucosal structure as a gel-like component of the mucosa
	alpha-2-antiplasmin (*Homo sapiens*)	endopeptidase inhibitor activity, protease binding, protein homodimerization activity, serine-type endopeptidase inhibitor activity
	L-xylulose reductase (*Mesocricetus auratus*)	identical protein binding, L-xylulose reductase (NADP+) activity, oxidoreductase activity, acting on NAD(P)H, quinone or similar compound as acceptor
	Abl interactor 1 (*Homo sapiens*)	cadherin binding, cytoskeletal protein binding, protein tyrosine kinase activator activity, SH3 domain binding, signaling adaptor activity
	IST1 homolog (*Bos taurus*)	ESCRT-III-like protein involved in cytokinesis, nuclear envelope reassembly and endosomal tubulation (By similarity). Is required for efficient abscission during cytokinesis (By similarity). Involved in recruiting VPS4A and/or VPS4B to the midbody of dividing cells (By similarity). During late anaphase, involved in

	nuclear envelope reassembly and mitotic spindle disassembly together with the ESCRT-III complex: IST1 acts by mediating the recruitment of SPAST to the nuclear membrane, leading to microtubule severing (By similarity). Recruited to the reforming nuclear envelope (NE) during anaphase by LEMD2 (By similarity). Regulates early endosomal tubulation together with the ESCRT-III complex by mediating the recruitment of SPAST (By similarity)
heme-binding protein 2 (*Mus musculus*)	heme binding
serotransferrin-A (*Xenopus laevis*)	metal ion binding
phosducin-like protein 3 (*Rattus norvegicus*)	protein folding chaperone, vascular endothelial growth factor receptor 2 binding
biglycan (*Xenopus laevis*)	May be involved in collagen fiber assembly

[1] The functions were identified based on Uniprot or NCBI database.

Table S2. The 49 unique proteins with high confidence for distinguishing legal species and other two species.

Protein name	Unique Peptides	MW [KD]	Isoelectric point
elongation factor 1-alpha 2 (*Rattus norvegicus*)	1	46.2	8.94
GTP cyclohydrolase 1 feedback regulatory protein (*Xenopus tropicalis*)	2	10.0	5.83
nicotinamide N-methyltransferase (*Homo sapiens*)	2	24.0	6.71
cathepsin D (*Chionodraco hamatus*)	3	89.4	5.31
speckle targeted PIP5K1A-regulated poly(A) polymerase (*Xenopus tropicalis*)	3	146.1	5.66
dihydrolipoyllysine-residue succinyltransferase component of 2-oxoglutarate dehydrogenase complex, mitochondrial (*Bos taurus*)	2	41.8	6.44
cystathionine beta-synthase (*Macaca fascicularis*)	3	61.6	7.44
calcyclin-binding protein (*Pongo abelii*)	4	19.0	6.60
bifunctional purine biosynthesis protein PURH (*Pongo abelii*)	3	53.3	6.67
DnaJ homolog subfamily C member 12 (*Homo sapiens*)	2	23.0	5.38
collagenase 3 (Fragment) (*Xenopus laevis*)	3	93.6	5.92
peptidyl-prolyl cis-trans isomerase D (*Rattus norvegicus*)	3	38.6	6.32

cytochrome c oxidase subunit 5A, mitochondrial (*Saguinus labiatus*)	3	22.8	9.31
3-hydroxyacyl-CoA dehydrogenase type-2 (*Bos taurus*)	2	27.2	8.72
protein POF1B (*Mus musculus*)	3	91.9	8.00
putative methyltransferase DDB_G0268948 (*Dictyostelium discoideum*)	1	22.4	7.85
isopentenyl-diphosphate Delta-isomerase 1 (*Mesocricetus auratus*)	2	26.4	6.00
60S ribosomal protein L22 (*Xenopus tropicalis*)	2	8.40	10.24
extracellular superoxide dismutase [Cu-Zn] (*Oryctolagus cuniculus*)	2	58.7	7.56
60S ribosomal protein L7 (*Gallus gallus*)	2	25.7	10.93
aldehyde oxidase 1 (*Oryctolagus cuniculus*)	1	131.1	6.73
40S ribosomal protein S27-like (*Bos taurus*)	1	11.5	9.13
COP9 signalosome complex subunit 8 (*Xenopus laevis*)	2	17.1	4.56
60S ribosomal protein L14 (*Mus musculus*)	1	23.8	10.42
protein ERGIC-53 (*Rattus norvegicus*)	2	69.3	4.97
ATPase asna1 (*Xenopus tropicalis*)	1	33.5	5.33
actin-related protein 2/3 complex subunit 1A (*Homo sapiens*)	1	37.6	8.41
vacuolar protein sorting-associated protein 37B (*Homo sapiens*)	1	31.7	6.57
TATA-binding protein-associated factor 2N (*Homo sapiens*)	1	40.5	4.83
mimecan (*Bos taurus*)	2	33.7	6.43
NAD(P) transhydrogenase, mitochondrial (*Homo sapiens*)	2	89.7	8.06
cytosolic purine 5'-nucleotidase (*Xenopus tropicalis*)	2	60.0	7.06
serine/threonine-protein kinase OSR1 (*Pongo abelii*)	1	52.1	8.27
V-type proton ATPase subunit d 1 (*Danio rerio*)	1	28.6	5.24
actin-related protein 2/3 complex subunit 3 (*Homo sapiens*)	1	21.6	8.35
MICOS complex subunit Mic19 (*Mus musculus*)	1	25.0	7.80
NAD-dependent malic enzyme, mitochondrial (*Mus musculus*)	1	66.8	7.56
NADH dehydrogenase [ubiquinone] 1 alpha subcomplex subunit 10, mitochondrial (*Homo sapiens*)	1	32.1	5.94

endoplasmic reticulum resident protein 44 (*Homo sapiens*)	1	50.9	5.30
enoyl-CoA hydratase domain-containing protein 2, mitochondrial (*Bos taurus*)	1	28.3	9.19
cytochrome P450 2C3 (*Oryctolagus cuniculus*)	1	50.9	8.91
ATP-binding cassette sub-family E member 1 (*Mus musculus*)	1	58.4	7.99
thioredoxin domain-containing protein 9 (*Bos taurus*)	1	25.9	7.03
complement component 1 Q subcomponent-binding protein, mitochondrial (*Rattus norvegicus*)	1	27.5	4.93
transmembrane and immunoglobulin domain-containing protein 1 (*Bos taurus*)	1	27.3	7.66
GTP cyclohydrolase 1 (*Gallus gallus*)	1	25.5	7.62
galectin-4 (*Bos taurus*)	1	62.5	5.71
phytanoyl-CoA dioxygenase domain-containing protein 1 (*Homo sapiens*)	1	20.8	6.15
gamma-crystallin-3 (*Xenopus laevis*)	1	25.2	7.94

Table S3. The 22 unique proteins with high confidence for distinguishing BgC and other three species.

Protein name	Unique Peptides	MW [KD]	Isoelectric point
cellular retinoic acid-binding protein 2 (*Xenopus laevis*)	2	15.9	7.80
peroxiredoxin (*Cynops pyrrhogaster*)	2	22.3	6.79
vacuolar protein sorting-associated protein 4A (*Rattus norvegicus*)	1	87.4	7.28
ATP synthase subunit g, mitochondrial (*Homo sapiens*)	2	11.5	9.54
aminoacyl tRNA synthase complex-interacting multifunctional protein 1 (*Homo sapiens*)	2	36.5	5.96
NAD-dependent protein deacylase sirtuin-5, mitochondrial (*Xenopus tropicalis*)	1	32.2	8.03
phosphatidylinositol 5-phosphate 4-kinase type-2 gamma (*Xenopus tropicalis*)	1	49.1	8.27
pyruvate dehydrogenase E1 component subunit alpha, somatic form, mitochondrial (*Bos taurus*)	2	40.2	8.07
ferrochelatase, mitochondrial (*Xenopus laevis*)	1	46.3	8.65
crk-like protein (*Homo sapiens*)	1	33.6	6.44
copper chaperone for superoxide dismutase (*Mus musculus*)	2	27.1	5.57

NADH dehydrogenase [ubiquinone] iron-sulfur protein 3, mitochondrial (*Mus musculus*)	1	30.0	7.72
aldehyde oxidase 1 (*Bos taurus*)	2	335.3	6.64
mitogen-activated protein kinase 12 (*Homo sapiens*)	2	41.8	6.47
probable ubiquitin carboxyl-terminal hydrolase FAF-X (*Homo sapiens*)	1	300.1	6.24
serine/threonine-protein kinase RIO3 (*Homo sapiens*)	1	55.8	5.62
small nuclear ribonucleoprotein F (*Xenopus laevis*)	1	7.70	4.41
eukaryotic translation initiation factor 3 subunit B (*Xenopus laevis*)	1	73.6	6.15
E3 ubiquitin-protein ligase KCMF1 (*Xenopus laevis*)	1	41.9	5.45
diacylglycerol O-acyltransferase 2 (*Xenopus tropicalis*)	1	17.5	9.20
ubiquitin-like modifier-activating enzyme 5 (*Xenopus laevis*)	1	41.8	5.60
methylglutaconyl-CoA hydratase, mitochondrial (*Mus musculus*)	1	27.0	9.26

Table S4. The 86 unique proteins with high confidence for distinguishing BmS and other three species.

Protein name	Unique Peptides	MW [KD]	Isoelectric point
tubulin alpha chain (*Xenopus laevis*)	1	41.9	4.86
heat shock 70 kDa protein (*Xenopus laevis*)	1	51.3	5.41
peroxisomal bifunctional enzyme (*Danio rerio*)	6	81.1	9.07
Myosin-4 (*Sus scrofa*)	2	239.6	5.88
ATP-binding cassette sub-family D member 3 (*Homo sapiens*)	3	70.9	9.70
myelin P2 protein (*Oryctolagus cuniculus*)	4	14.0	6.55
40S ribosomal protein S7 (*Xenopus laevis*)	5	27.5	9.96
vesicle-associated membrane protein-associated protein A (*Bos taurus*)	1	25.6	8.40
40S ribosomal protein S17 (*Bos taurus*)	2	8.90	5.49
protein transport protein Sec31A (*Rattus norvegicus*)	3	121.7	5.78
phosphoglycerate mutase 2 (*Homo sapiens*)	1	25.5	7.17

pyruvate carboxylase, mitochondrial (*Homo sapiens*)	3	93.1	6.67
secretory carrier-associated membrane protein 2 (*Homo sapiens*)	1	35.2	6.20
lambda-crystallin homolog (*Homo sapiens*)	2	140.5	3.81
mitochondrial import inner membrane translocase subunit Tim13-A (*Xenopus laevis*)	2	10.8	8.48
S-methyl-5'-thioadenosine phosphorylase (*Xenopus tropicalis*)	2	43.1	6.79
lamin-L(I) (*Xenopus laevis*)	1	69.2	5.02
AP-3 complex subunit delta-1 (*Homo sapiens*)	1	148.2	8.53
transcobalamin-2 (*Homo sapiens*)	2	37.1	6.90
protein NipSnap homolog 1 (*Mus musculus*)	2	27.8	9.09
D-beta-hydroxybutyrate dehydrogenase, mitochondrial (*Rattus norvegicus*)	1	39.0	9.17
protein HID1 (*Mus musculus*)	2	73.6	6.71
adenylate kinase 2, mitochondrial (*Xenopus tropicalis*)	2	26.3	6.44
ester hydrolase C11orf54 homolog (*Xenopus laevis*)	1	32.1	6.95
ATP-dependent 6-phosphofructokinase, muscle type (*Bos taurus*)	1	79.8	8.87
myristoylated alanine-rich C-kinase substrate (*Gallus gallus*)	1	28.6	4.46
pirin (*Homo sapiens*)	1	33.8	6.58
heme-binding protein 2 (*Homo sapiens*)	1	23.6	7.84
elongation factor 1-beta (*Xenopus laevis*)	1	42.8	5.39
keratin, type II cytoskeletal 75 (*Homo sapiens*)	1	56.0	6.58
60S ribosomal protein L10 (*Macaca fascicularis*)	2	25.5	9.35
protein CDV3 homolog (*Xenopus tropicalis*)	1	21.8	5.31
succinate--CoA ligase [ADP/GDP-forming] subunit alpha, mitochondrial (*Rattus norvegicus*)	1	38.1	9.39
eukaryotic peptide chain release factor GTP-binding subunit ERF3A (*Mus musculus*)	1	58.4	4.93
ovoinhibitor (*Gallus gallus*)	1	28.2	6.86
charged multivesicular body protein 1b (*Xenopus laevis*)	2	21.5	7.87
elongin-B (*Rattus norvegicus*)	2	12.7	5.11

2-amino-3-ketobutyrate coenzyme A ligase, mitochondrial (*Homo sapiens*)	1	36.9	7.30
7-methylguanosine phosphate-specific 5'-nucleotidase A (*Xenopus laevis*)	1	30.9	6.54
N-acylneuraminate cytidylyltransferase (*Pongo abelii*)	1	44.4	6.60
ras-related protein Ral-A (*Saguinus oedipus*)	1	23.5	7.80
parvalbumin beta (*Xenopus laevis*)	1	15.3	4.75
electron transfer flavoprotein subunit beta (*Pongo abelii*)	1	24.1	8.51
selenoprotein O (*Mus musculus*)	1	67.3	5.57
regulator of microtubule dynamics protein 2 (*Xenopus tropicalis*)	1	47.0	5.94
charged multivesicular body protein 4c (*Xenopus laevis*)	1	25.3	6.19
mitochondrial proton/calcium exchanger protein (*Xenopus tropicalis*)	1	54.9	5.07
eukaryotic peptide chain release factor subunit 1 (*Xenopus tropicalis*)	1	44.2	8.09
hippocalcin-like protein 1 (*Taeniopygia guttata*)	1	22.7	5.49
60S ribosomal protein L5-A (*Xenopus laevis*)	1	34.2	9.67
transmembrane emp24 domain-containing protein 2 (Fragment) (*Cricetulus griseus*)	1	23.8	5.02
inositol monophosphatase 3 (*Xenopus laevis*)	1	35.5	7.90
dolichyl-diphosphooligosaccharide-protein glycosyltransferase subunit STT3B (*Canis lupus familiaris*)	1	88.5	8.66
cystatin-2 (*Crotalus adamanteus*)	1	9.90	9.20
actin-related protein 2/3 complex subunit 5 (*Mus musculus*)	1	16.9	5.55
core histone macro-H2A.1 (*Rattus norvegicus*)	1	39.6	9.57
plastin-3 (*Bos taurus*)	1	59.8	5.55
semaphorin-3C (*Gallus gallus*)	1	70.0	7.47
alpha-mannosidase 2C1 (*Mus musculus*)	1	94.2	6.29
putative nascent polypeptide-associated complex subunit alpha-like protein (*Homo sapiens*)	1	198.3	4.56
ubiquitin-like protein 4A (*Xenopus tropicalis*)	1	8.30	4.92
hemoglobin subunit alpha-5 (*Xenopus laevis*)	1	18.1	7.21
signal transducing adapter molecule 2 (*Homo sapiens*)	1	54.9	5.24

lysozyme C (*Opisthocomus hoazin*)	1	14.2	6.74
transmembrane emp24 domain-containing protein 4 (*Mus musculus*)	1	18.6	8.47
amyloid-like protein 2 (*Homo sapiens*)	2	88.9	5.17
mesencephalic astrocyte-derived neurotrophic factor (*Bos taurus*)	2	20.2	8.48
inter-alpha-trypsin inhibitor heavy chain H3 (*Oryctolagus cuniculus*)	1	109.2	7.42
complement factor H (*Bos taurus*)	1	50.3	7.09
testis development-related protein (*Xenopus laevis*)	1	32.9	5.30
ATP-binding cassette sub-family F member 1 (*Homo sapiens*)	1	92.4	6.40
MAP7 domain-containing protein 2 (*Mus musculus*)	1	110	8.22
laminin subunit beta-2 (*Rattus norvegicus*)	1	172	7.75
guanosine-3',5'-bis(diphosphate) 3'-pyrophosphohydrolase MESH1 (*Xenopus tropicalis*)	1	15.4	7.20
arylsulfatase D (*Homo sapiens*)	1	21.0	8.72
phosphomevalonate kinase (*Bos taurus*)	1	24.0	5.58
microtubule-associated protein RP/EB family member 2 (*Xenopus laevis*)	1	37.0	5.85
galectin-4 (*Sus scrofa*)	1	29.5	9.51
maturin (*Xenopus tropicalis*)	1	15.0	4.17
oxysterol-binding protein-related protein 2 (*Homo sapiens*)	1	67.7	7.15
protein QNR-71 (*Coturnix japonica*)	1	57.2	5.41
hemoglobin subunit alpha-3 (*Xenopus tropicalis*)	1	15.5	6.52
nuclease-sensitive element-binding protein 1 (*Xenopus laevis*)	2	57.3	9.38
protein of unknown function	1	22.1	9.94
protein of unknown function	1	13.5	6.76
protein of unknown function	1	37.9	8.95

Table S5. The 5 unique proteins with high confidence for distinguishing BrS and other three species.

Protein name	Unique Peptides	MW [KD]	Isoelectric point
small nuclear ribonucleoprotein Sm D3 (*Xenopus laevis*)	1	13.9	10.32
cofilin-2 (*Xenopus tropicalis*)	1	30.9	7.62
serum paraoxonase/arylesterase 2 (*Meleagris gallopavo*)	1	22.4	6.37
ATPase family AAA domain-containing protein 3-B (*Xenopus laevis*)	1	66.5	8.68
biglycan (*Xenopus laevis*)	1	34.3	9.29

Table S6. The 60 unique proteins with high confidence for distinguishing BaS and other three species.

Protein name	Unique Peptides	MW [KD]	Isoelectric point
mannose-6-phosphate isomerase (*Mus musculus*)	3	46.0	5.53
putative methyltransferase DDB_G0268948 (*Dictyostelium discoideum*)	2	22.7	6.42
S-formylglutathione hydrolase (*Sus scrofa*)	2	32.0	6.27
probable threonine-tRNA ligase 2, cytoplasmic (*Xenopus tropicalis*)	3	108.4	6.80
short-chain specific acyl-CoA dehydrogenase, mitochondrial (*Mus musculus*)	3	38.4	7.71
nuclear factor 7, ovary (*Xenopus laevis*)	3	63.2	6.49
eukaryotic translation initiation factor 2 subunit 3 (*Gallus gallus*)	3	47.1	8.32
LIM domain only protein 7 (*Homo sapiens*)	3	228	6.57
THAP domain-containing protein 4 (*Mus musculus*)	1	18.6	6.62
prosaposin (*Bos taurus*)	3	60.7	5.39
CTP synthase 1-A (*Xenopus laevis*)	2	66.1	6.86
catenin beta-1 (*Homo sapiens*)	2	83.7	5.63
bis(5'-nucleosyl)-tetraphosphatase [asymmetrical] (*Homo sapiens*)	2	17.1	5.81
coatomer subunit gamma-1 (*Homo sapiens*)	2	85.9	5.31
interferon-induced, double-stranded RNA-activated protein kinase (*Rattus norvegicus*)	1	59.4	9.17
methionine-tRNA ligase, cytoplasmic (*Xenopus laevis*)	2	87.4	7.20

D-tyrosyl-tRNA (Tyr) deacylase 1 (*Homo sapiens*)	1	27.6	6.70
PRKCA-binding protein (*Rattus norvegicus*)	1	36.7	5.30
P2X purinoceptor 4 (*Homo sapiens*)	1	43.0	7.49
lamin-L(II) (*Xenopus laevis*)	3	62.5	5.59
pleckstrin homology domain-containing family A member 1 (*Homo sapiens*)	1	40.6	7.09
phenylalanine-tRNA ligase beta subunit (*Pongo abelii*)	2	45.2	8.31
dynein light chain 2, cytoplasmic (*Rattus norvegicus*)	2	10.3	7.37
prolyl endopeptidase (*Sus scrofa*)	2	79.6	6.40
trifunctional purine biosynthetic protein adenosine-3 (*Gallus gallus*)	1	103.3	5.74
N-acetylgalactosamine kinase (*Homo sapiens*)	2	49.6	5.77
serine/arginine-rich splicing factor 9 (*Rattus norvegicus*)	2	25.5	9.57
ectonucleotide pyrophosphatase/phosphodiesterase family member 7 (*Rattus norvegicus*)	1	40.5	8.35
bifunctional coenzyme A synthase (*Sus scrofa*)	2	61.1	5.50
palmdelphin (*Xenopus laevis*)	1	54.8	5.31
disintegrin and metalloproteinase domain-containing protein 10 (*Xenopus laevis*)	1	84.7	7.30
CDGSH iron-sulfur domain-containing protein 1 (*Mus musculus*)	1	10.9	8.66
pyruvate dehydrogenase E1 component subunit beta, mitochondrial (*Bos taurus*)	1	30.4	6.39
1,4-alpha-glucan-branching enzyme (*Equus caballus*)	1	70.5	6.25
putative Kunitz-type serine protease inhibitor (*Austrelaps labialis*)	1	41.9	5.16
casein kinase II subunit alpha (*Xenopus laevis*)	2	46.5	8.27
ras-related protein Rab-9B (*Pongo abelii*)	1	49.8	6.79
SEC23-interacting protein (*Homo sapiens*)	2	108.7	5.33
galactose-1-phosphate uridylyltransferase (*Mus musculus*)	1	45.9	4.93
ubiquitin carboxyl-terminal hydrolase MINDY-1 (*Bos taurus*)	1	37.5	4.48
calcium-binding mitochondrial carrier protein SCaMC-1-B (*Xenopus laevis*)	1	75.2	8.66
vacuolar protein-sorting-associated protein 25 (*Xenopus laevis*)	1	25.1	8.46

hemoglobin subunit alpha-5 (*Xenopus laevis*)	1	13.3	6.52
PDZ and LIM domain protein 5 (*Homo sapiens*)	1	66.3	8.05
TBC1 domain family member 13 (*Homo sapiens*)	1	37.2	5.44
superoxide dismutase [Cu-Zn] (*Xenopus tropicalis*)	1	11.0	6.92
programmed cell death protein 10 (*Xenopus laevis*)	1	24.4	7.39
asparagine--tRNA ligase, cytoplasmic (*Mus musculus*)	1	47.9	5.26
stromelysin-2 (*Homo sapiens*)	1	85.3	9.50
dynactin subunit 1 (*Xenopus laevis*)	1	116.8	5.19
golgi apparatus protein 1 (*Gallus gallus*)	1	122	6.95
epidermal growth factor receptor substrate 15 (*Homo sapiens*)	1	104.7	4.67
F-box only protein 7 (*Homo sapiens*)	1	52.0	7.21
E3 ubiquitin-protein ligase UBR4 (*Homo sapiens*)	1	275.7	6.48
vacuolar protein sorting-associated protein 35 (*Mus musculus*)	1	95.6	6.19
cytosolic acyl coenzyme A thioester hydrolase (*Rattus norvegicus*)	1	14.8	8.78
sideroflexin-1 (*Sus scrofa*)	1	29.0	8.57
serine/threonine-protein phosphatase CPPED1 (*Xenopus tropicalis*)	1	42.1	5.29
long-chain-fatty-acid-CoA ligase 1 (*Mus musculus*)	1	72.4	7.20
E3 ubiquitin-protein ligase HECTD3 (*Mus musculus*)	1	80.8	5.45

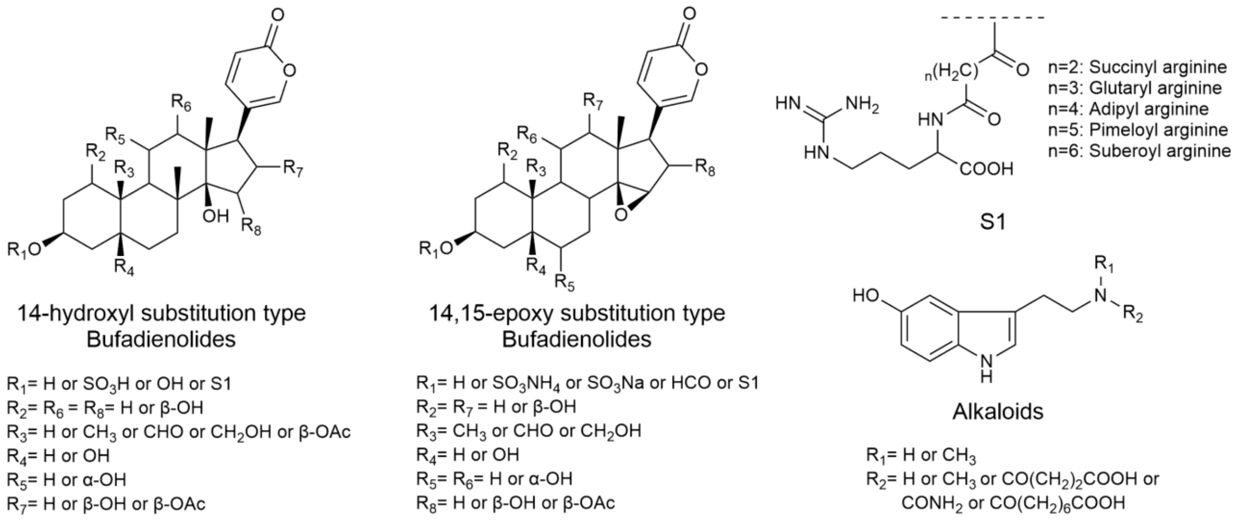

Figure S1. The typical structures and substitutions of micromolecules in TV.

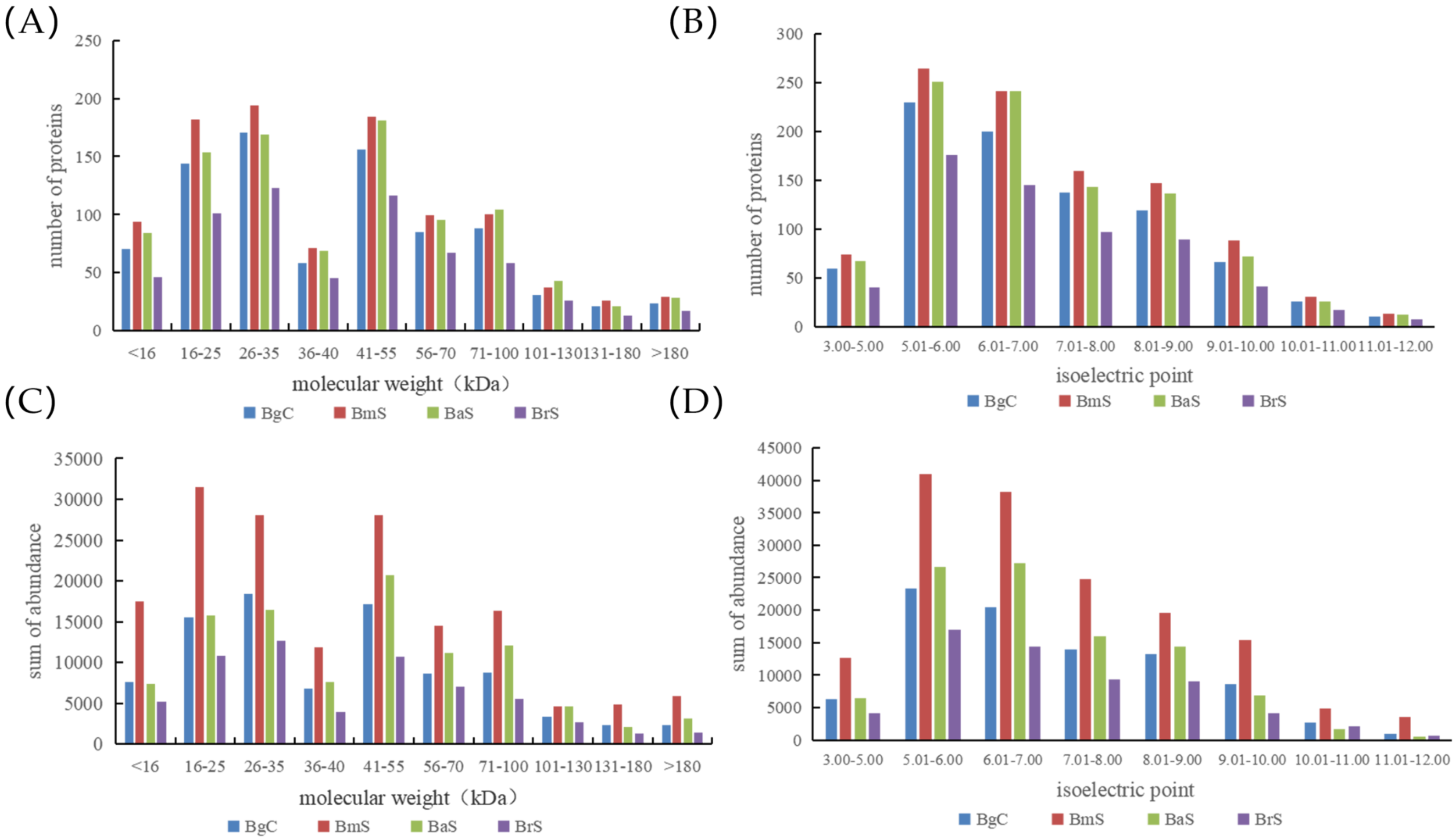
(A)
number of proteins
molecular weight（kDa）
<16
16-25
26-35
36-40
41-55
56-70
71-100
101-130
131-180
>180
BgC
BmS
BaS
BrS
(B)
number of proteins
isoelectric point
3.00-5.00
5.01-6.00
6.01-7.00
7.01-8.00
8.01-9.00
9.01-10.00
10.01-11.00
11.01-12.00
(C)
sum of abundance
molecular weight（kDa）
(D)
sum of abundance
isoelectric point

Figure S2: The distribution of proteins in molecular weights and isoelectric point for four Bufo species, (A) The number of proteins in each molecular weight range. (B) The number of proteins in each isoelectric point range. (C) The sum of abundance of all proteins in each molecular weight range. (D) The sum of abundance of all proteins in each isoelectric point range
